# Discovery of a new gall-inducing species, *Aciurinaluminaria* (Insecta, Diptera, Tephritidae) via multi-trait integrative taxonomy

**DOI:** 10.3897/zookeys.1214.130171

**Published:** 2024-10-07

**Authors:** Quinlyn Baine, Branden White, Vincent G. Martinson, Ellen O. Martinson

**Affiliations:** 1 Department of Biology, University of New Mexico, 219 Yale Blvd, Albuquerque, NM 87131, USA University of New Mexico Albuquerque United States of America

**Keywords:** *Bigeloviae*, candle, ddRAD, *Ericameria*, flame, marshmallow, *nauseosa*, rabbitbrush, tephritid, *trixa*, wing

## Abstract

Integrative taxonomic practices that combine multiple lines of evidence for species delimitation greatly improve our understanding of intra- and inter-species variation and biodiversity. However, extended phenotypes remain underutilized despite their potential as a species-specific set of extracorporeal morphological and life history traits. Primarily relying on variations in wing patterns has caused taxonomic confusion in the genus *Aciurina*, which are gall-inducing flies on Asteraceae plants in western North America. However, species display distinct gall morphologies that can be crucial for species identification. Here we investigate a unique gall morphotype in New Mexico and Colorado that was previously described as a variant of that induced by *Aciurinabigeloviae* (Cockerell, 1890). Our analysis has discovered several consistent features that distinguish it from galls of *A.bigeloviae*. A comprehensive description of *Aciurinaluminaria* Baine, **sp. nov.** and its gall is provided through integrative taxonomic study of gall morphology, host plant ecology, wing morphometrics, and reduced-representation genome sequencing.

## Introduction

Species delimitation is an essential step in our collective goal as biologists to calculate the total diversity of life on the planet (Dayrat 2005), and is particularly vital in the midst of ongoing decline of insects – the world’s most species-rich group of animals (Dirzo et al. 2014; Stork et al. 2015; Sánchez-Bayo and Wyckhuys 2019). However, species divisions are frequently unclear, particularly where purely morphological descriptions include high levels of intra-species variation, which poses a significant challenge in taxonomy, as it can result in unreliable diagnosis of species boundaries (Gentile et al. 2021). Most descriptions of insect species are made from adult morphological characters alone and are presented as a list of traits that have some author-determined significance in recognition (e.g., coloration, wing patterns, integumental texture). This issue can be addressed by combining multiple lines of evidence, such as morphological, ecological, and geographical data, to make more accurate and robust species delimitations; a long-standing practice coined in recent decades as “integrative taxonomy” (Schlick-Steiner et al. 2010). Taxonomists are also now equipped with molecular tools that can provide deep genome-wide datasets to investigate intra-taxon distinctions, even in cryptic or rare groups of arthropods (Hebert et al. 2003; Sheikh et al. 2022). An integrative taxonomy approach to species description improves our overall species estimates and identification of significant radiations in evolutionary history. By embracing a holistic approach, integrative taxonomy allows for a more nuanced understanding of biodiversity, leading to more precise species identification (Schlick-Steiner et al. 2010). This not only enhances our knowledge of the natural world but also is crucial for conservation efforts, as accurately identifying species is foundational to protecting them and their habitats.

Though many have adopted integrative taxonomic description, a potentially powerful tool for species delimitation remains under-utilized: the extended phenotype. This refers to an organism’s genetic expression that can be observed beyond their own bodies, particularly in the case of animals that construct or modify unique structures such as bird nests and spider webs (Blamires 2013; Mainwaring et al. 2014). These extensions of the phenotype represent species-specific behaviors and adaptations, establishing them as a key piece of the species’ ecology, and a set of additional morphological traits that can be used in species delimitation (Bailey et al. 2009; Freudenstein et al. 2016). For example, gall-inducing insects create structures that are so frequently species-specific that they can be used as a diagnostic character for identification (Raman et al. 2005; Bailey et al. 2009; Redfern 2011; Russo 2021). Integrating extended phenotypes in species description will likely enhance the resolution of taxonomic classifications, especially with ecosystem engineers like gall-inducing insects.

The genus *Aciurina* (Diptera: Tephritidae) are gall-inducing flies on Asteraceae shrubs in western North America (Foote et al. 1993). Many species in this genus are informally recognized by gall morphological characters, and, similarly to many tephritid “picture-wing” flies, formally identified with diagnostic black and transparent markings of the wings. The common and widespread species *Trypetabigeloviae* was first described only as a “white, woolly [sic], and conspicuous” gall on the plant *Bigelovia* (Cockerell 1890a). The fly was then described in the same year as both *T.bigeloviae* and T.bigeloviaevar.disrupta based on a single distinction in the postero-distal hyaline region of the wing: in *disrupta* this area is divided (disrupted) into two by a complete black marking (Cockerell 1890b). Bates (1935) later re-assigned *T.bigeloviae* to the genus *Aciurina*, including the variety *disrupta* which he did not consider distinct, citing variation of this character in specimens from the same locality. Wing marking variation led Steyskal (1984) in his revision of *Aciurina* to then synonymize the type species *Aciurinatrixa* Curran, 1932 and *Aciurinasemilucida* Bates, 1935 with *Aciurinabigeloviae* (Cockerell, 1890), which he characterized as being the most variable species of the genus. However, Dodson and George (1986) soon after described the likely recently diverged relationship of *A.bigeloviae* and *A.trixa* based on thorough examination of gall morphology, host plant ecology, hybrid breeding success, and genetic allelic frequencies. First Goeden and Teerink (1996) reinstated *A.semilucida*, then Headrick et al. (1997) officially reinstated *A.trixa* as a species distinct from *A.bigeloviae* and provided a larval description. The most reliable diagnostic character established between the sister species was gall morphology: *A.bigeloviae* has a white, wooly “cotton” gall and *A.trixa* has a resinous, waxy “smooth” gall. Both species form galls on *Ericamerianauseosa* (Pall. ex Pursh) G.L.Nesom & G.I.Baird, however on different varieties of the species.

**Figure 1.**
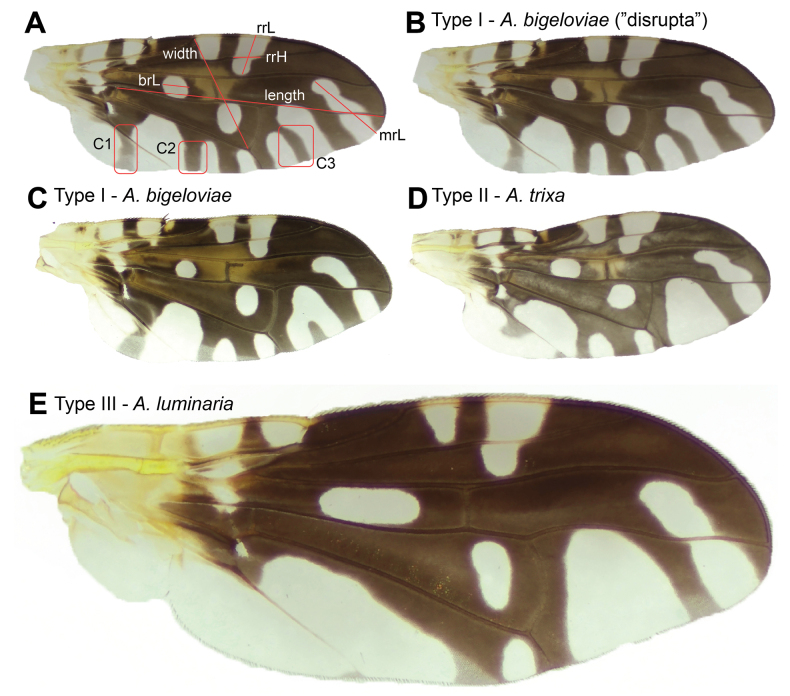
Wing morphotypes and measurements taken **A** diagram of characters and measurements defined and used in analysis **B***A.bigeloviae***C***A.bigeloviae***D***A.trixa***E***A.luminaria* sp. nov. Not to scale.

Dodson and George (1986) also described in detail the confusing variation in wing patterns noted by the other authors above by establishing three pattern categories among the two species: 1) the Type I pattern that included the originally described wing pattern for A.bigeloviae plus that of var. disrupta, 2) the Type II pattern which matched the description of *A.trixa*, and 3) the Type II’ pattern (hereafter referred to as Type III) as a modified version of the *A.trixa* wing pattern, but from flies reared from *A.bigeloviae*-type cotton galls (Fig. 1). The authors admit that this third pattern, with its unexpected gall-wing morphological pairing, left them somewhat stumped: “Whether they belong to *bigeloviae*, *trixa*, or a third species probably will not be resolved until further studies parallel to those reported here are carried out” (Dodson and George 1986).

Here we follow this thread and using an integrative taxonomic approach that employs gall morphology, previously unexplored host plant ecology, extensive wing morphometric and character analysis, and multi-locus reduced representation genome sequencing, provide evidence that the Type III flies are a third species. We provide a name for this species, *Aciurinaluminaria*, and a complete morphological description of the adult fly and its gall.

## Materials and methods

**Figure 2.**
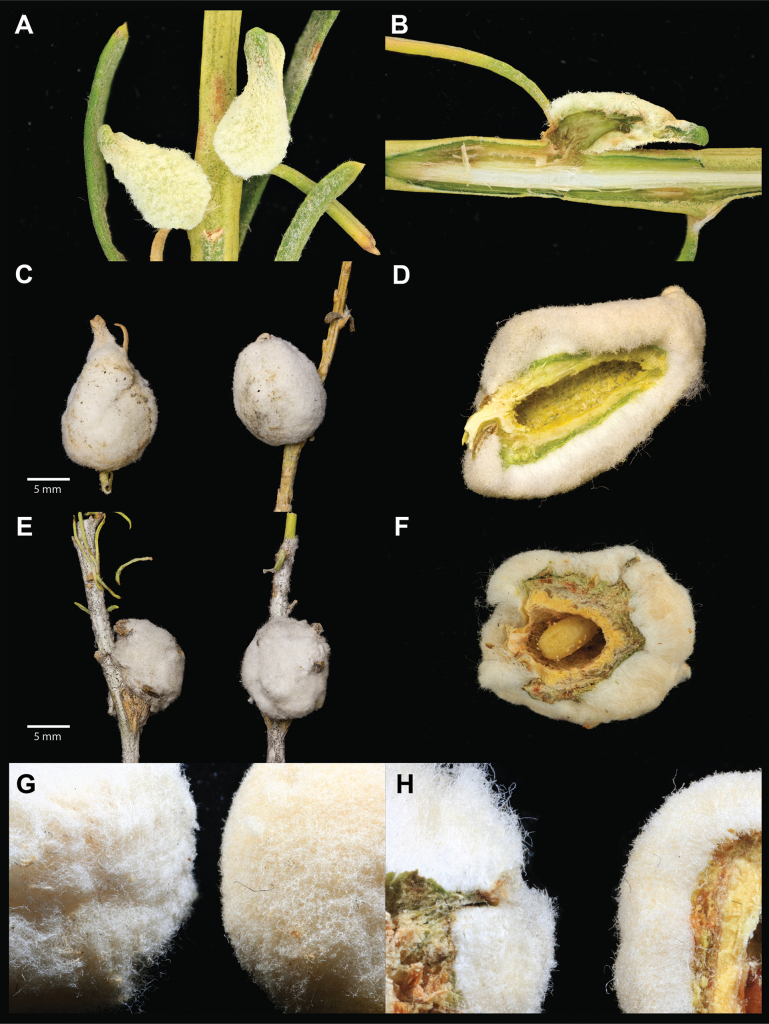
Galls of *A.luminaria* and *A.bigeloviae***A** immature *A.luminaria* galls **B** internal view of immature *A.luminaria* gall with early instar larva burrowing into the stem while gall develops **C** mature *A.luminaria* galls **D** internal view of mature *A.luminaria* gall with full-sized larval chamber **E** mature *A.bigeloviae* galls **F** internal view of *A.bigeloviae* gall with a mature larva in the larval chamber. Side by side comparison of *A.bigeloviae* (left) and *A.luminaria* (right) tomentum texture and uniformity **G** external view **H** internal view.

We observed in previous collections of *A.bigeloviae* that “cotton” galls in New Mexico could be categorized into two groups by general gall shape: spherical and teardrop-shaped (Fig. 2). We were able to confirm from rearing haphazardly collected galls that the spherical cotton galls were induced by flies with Type I wing morphology (*A.bigeloviae*), and the teardrop-shaped galls were induced by flies with Type III wing morphology. The ability to recognize the different gall morphs in the field allowed us to perform targeted collections for each morphotype.

We systematically collected and reared *A.bigeloviae* and *A.trixa* galls in New Mexico between 2021–2022 following methods outlined in Baine et al. (2023a). Type III galls were collected haphazardly from identified populations throughout New Mexico and Colorado in April and May of 2021–2023 by clipping sections of stem with gall attached and transporting them to the University of New Mexico. In the lab, galls were placed in insect rearing cages (BugDorm) and kept at 45% relative humidity. Adult flies were removed from cages as they emerged and preserved in 100% EtOH at -20 °C for the following analyses. A subset of pinned flies and galls was examined for morphological characters and photographed using an EOS 40D camera fitted with a 65 mm MP-E macro photo lens (Canon) mounted on Stackshot macro rail with controller (Cognisys), and then focus stacked with Zerene Stacker software. Representative flies and galls of each sampled population, plus other material examined, including the holotype of *A.luminaria*, were deposited in the following collections: The Museum of Southwestern Biology, Arthropods Division, New Mexico (**MSBA**); Smithsonian Institution, National Museum of Natural History, Washington DC (**USNM**); and William F. Barr Entomological Museum, Idaho (**WFBM**).

*Aciurina* species are frequently documented as specialists on particular varieties of *E.nauseosa*. For example, *A.bigeloviae* is associated with E.n.subsp.nauseosavar.graveolens, and *A.trixa* in New Mexico is associated with E.n.subsp.nauseosavar.latisquamea (Dodson and George 1986). To determine the host plant identity of Type III galls, we additionally returned to a subset of sites during the flowering season in the fall of 2022 and 2023 to obtain plant voucher specimens. Plant samples were identified using Anderson (2006) and Allred and Jercinovic (2020), and deposited in the MSB Herbarium.

### Wing morphology

A total of 62 female *Aciurina* specimens of the three morphotypes from 19 populations were selected for morphological assessment. Both wings of each specimen were carefully removed by pulling at the connection point where the tegula meets the thorax. Wings were mounted on glass slides with a Euparal mounting medium (Hempstead Halide). The edges of the cover slide were then sealed with clear nail polish and slides were left to dry at room temperature for 24 hours. Images of each wing were taken using a Axiocam 208 color camera mounted on a Stemi 508 microscope (Zeiss). Measurements were taken in ZEN 3.6 blue edition (Zeiss). The methodology used to take standardized proxy measurements of the wing width (represented by distance from apex of vein R1 to junction of vein M4 and crossvein dm-m) and length (represented by distance from junction of vein M4 and crossvein bm-m to apex of vein M4) follows Baleba et al. (2019). A total of seven different measurements and three different categorical observations were made for each wing (Fig. 1A). To facilitate comparison without influence of difference in overall body size, each measurement was divided by our measurement for wing length. Finally, measurements from each wing pair were averaged. We then compared morphotype III to both morphotype I and II using each set of measurements by analysis of variance (ANOVA, *aov*) or Kruskal-Wallis where assumptions for ANOVA were not met (*kruskal.test*), and each set of categorical variables by Pearson’s 𝛘^2^ test (*chi.test*) in R version 4.2.2 (R Core Team 2021).

The terminology we used for venation and cells follows Cumming and Wood (2017), and our selected measurements and categorical variables are defined as follows (Fig. 1A):

- **brL**: The maximum diameter of the subapical hyaline spot of cell br. This spot has a circular-elliptical shape, so the measurement typically follows a line from one elliptical-vertex to the other.
- **rrL**: The maximum width of the hyaline region located within cells r _1_ and r _2+3_, crossing vein R _2+3_: measurement taken from the midpoint of the vein C to the parabolic vertex of the shape. This pattern occasionally reaches vein R _4+5_; in this case the midpoint that borders this vein is used instead of a parabolic vertex.
- **rrH**: The maximum length of the same region hyaline in cells r _1_ and r _2+3_, measured along vein R _2+3_.
- **mrL**: The maximum length of the subapical hyaline region in cells r _4+5_ and m: length taken from the postero-distal corner to the parabolic vertex of the region.
- **C1**: The presence or absence of a complete medial stripe within the anal lobe from veins CuA+CuP to the posterior wing margin.
- **C2**: The presence or absence of a complete medial stripe within cell m _4_ from vein M _4_ to the posterior wing margin.
- **C3**: The extent of black medial stripe in the large subbasal hyaline region on the posterior margin of the wing within cell m. Three conditions exist: stripe absent, stripe incomplete, and stripe completely bisecting the region (var. *disrupta* morphology).

### Genomics

From three populations each of *A.bigeloviae*, *A.trixa* and Type III, we extracted whole-body DNA from three replicates (total n = 27) using the DNeasy Blood & Tissue kit and protocol (Qiagen), and quantified nucleic acid with a Qubit 3.0 fluorometer (Invitrogen). We generated genotypes for each sample from single-nucleotide polymorphisms (SNPs) derived from double digest restriction site associated DNA sequencing (ddRADseq) (Peterson et al. 2012). DNA was digested with enzymes EcoRI and MseI, and fragments were coupled with Illumina adaptors through T4 ligation. Pooled fragments were used for PCR with a proofreading enzyme (Iproof; BioRad), and fragment size selection for 300–450 bp was performed using a Pippin Prep quantitative electrophoresis unit. An Illumina NovaSeq S2 housed at the University of Texas at Austin Genomic Sequencing and Analysis Facility (Austin, TX) generated sequences of ~100 bp from input fragments. Trimmomatic 0.39 was used to truncate and filter demultiplexed single-end reads to a threshold of 85 bp (Bolger et al. 2014). Sequence files are deposited in NCBI GenBank under BioProject ID PRJNA1075688.

We mapped trimmed reads *de novo* using the Stacks 2.61 (Catchen et al. 2011) wrapper *denovo_map.pl* with the selected parameters: 5 minimum reads per stack, 3 maximum mismatches per locus, 3 maximum mismatches per stack, and minimum 80% individuals required per locus (parameter optimization was performed generally following Paris et al. 2017). We then filtered stacks in R with *SNPfiltR* (DeRaad 2022) and the settings: maximum depth of 50, maximum missing per sample 80%, minimum SNP completeness 80%, and minimum minor allele count of 1. Filtered SNP loci were used for principal component analysis (PCA) using packages *vcfR* (Knaus and Grünwald 2017), *dartR* (Gruber et al. 2018) and *adegenet* (Jombart 2008). We also calculated π for each population as a measure of nucleotide diversity. Structure was estimated using sparse nonnegative matrix factorization (sNMF) with package *LEA* (Frichot and Francois 2015). For each K value, where K represents the number of clusters ranging from 1 to 9, we performed 50 iterations. We then selected and graphed the best result from the most optimal K value, as identified through the cross-entropy criterion.

SNPs per sample were concatenated to generate sequences for each individual, and sequences were aligned in Stacks. Phylogeny was inferred by maximum likelihood (ML) tree with IQ-TREE 2.2.0 (Minh et al. 2020). ModelFinder (Kalyaanamoorthy et al. 2017) was used to select the best model as determined by Akaike Information Criterion (AIC). We calculated branch support from 1000 ultrafast bootstrap (UFBoot) (Hoang et al. 2018) and 1000 Shimodaira–Hasegawa approximate likelihood ratio test (SH-aLRT) (Guindon et al. 2010) replications. We report the selected model and basal branch support values. Sequence reads used in all above genomic analyses are deposited in NCBI GenBank within BioProject PRJNA1075688.

## Results

### Wing morphology

Morphometric comparisons of *A.bigeloviae* and *A.trixa* with the Type III morphotype highlighted regions of the wing that differ significantly in relative size and can therefore be used as diagnostic characters. The greater length of the hyaline spot within cell br (measurement brL) in Type III is the most notably distinct wing measurement from the other two morphotypes (*A.bigeloviae* 𝛘^2^ = 20.57, p < 0.0001; *A.trixa* 𝛘^2^ = 20.83, p < 0.0001). Type III also differs in the dimensions of the hyaline spot in r_1_ and r_2+3_, being longer than in *A.bigeloviae* (measurement rrH, F = 30.61, p < 0.0001), and wider than in *A.trixa* (measurement rrL, F = 7.99, p < 0.01). Finally, the measurement mrL is slightly greater in *A.trixa* than in Type III (F = 4.44, p < 0.05).

Despite variation, *A.bigeloviae* wings had the darkened stripes represented by the selected characters consistently present, while in Type III they were absent (Table 1). The absence of the dark stripe within the hyaline region in cell m (C3) that frequently separates *A.trixa* from *A.bigeloviae*, also separates the Type III morphotype from *A.bigeloviae*, but not from *A.trixa*. See Suppl. material 1: fig. S1 for wing character variation in each of the three morphotypes sampled.

**Table 1.** Significance values from 𝛘^2^ tests to compare the presence/absence of wing characters in the three morphotypes.

Wing pattern character	*A.bigeloviae* ~ Type III	*A.trixa* ~ Type III
C1	𝛘^2^ = 50.22, df = 1, p < 0.0001	𝛘^2^ = 36.29, df = 1, p < 0.0001
C2	𝛘^2^ = 68, df = 1, p <0.0001	𝛘^2^ = 65.33, df = 1, p < 0.0001
C3	𝛘^2^ = 47.28, df = 2, p < 0.0001	NS

### Genomics

**Figure 3.**
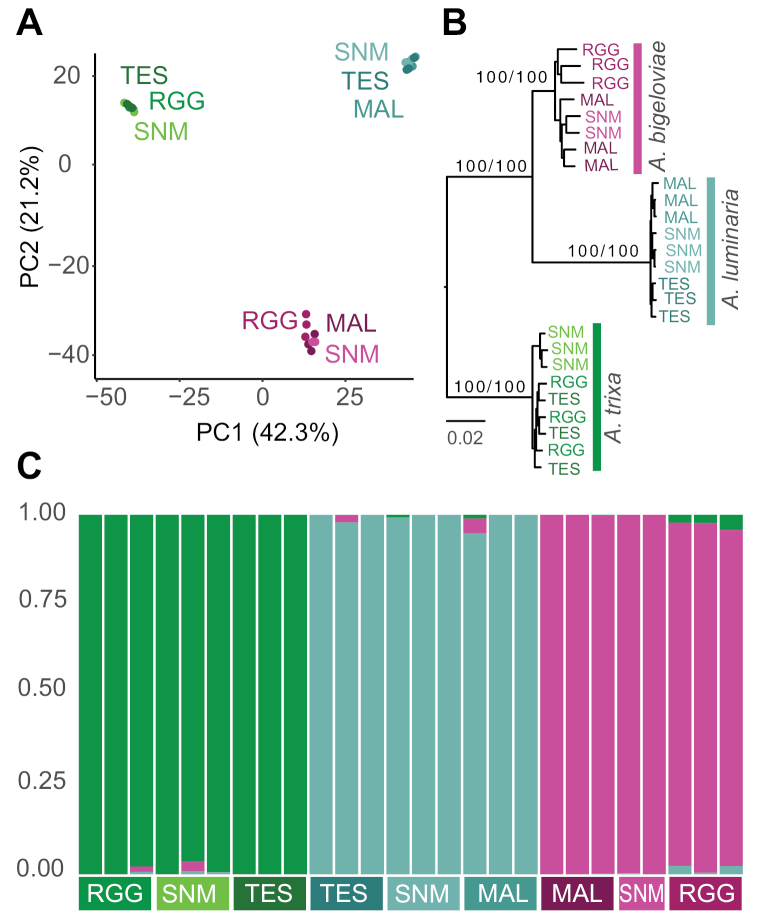
Results from SNP comparisons across populations (labeled as three-letter abbreviations) of the three morphotypes (labeled by color) **A**PCA plot **B** Maximum likelihood tree with basal support values UFBoot + SH-aLRT**C**sNMF structure-like admixture plot.

From the ddRAD sequencing of the three morphotypes, 42,838 SNPs were retained after filtering. A single sample was filtered out for high relative missingness, so we used 26 samples total for the following analyses. The first principal component (PC1) in the PCA performed accounted for 42.3% of the variance, splitting the morphotypes into discrete clusters that match to gall morphology (Fig. 3A). The second principal component (PC2) explained an additional 21.2% of the variance, splitting Type III from *A.bigeloviae*, and also with minimal loadings on geographic variation within *A.bigeloviae* populations (Fig. 3A). The PCA represents the greatest amount of variance exists between the three morphotypes. Average genetic diversity across all sites (π) was lower in Type III (0.0008) than in *A.bigeloviae* (0.0014) or *A.trixa* (0.0011) across all populations (0.0775, 0.1324, 0.1083, respectively across variant sites). Genetic diversity was also lower by a comparable margin in Type III across the populations at the sympatric site for all three morphotypes, “SNM” (0.0767 versus 0.12 *A.bigeloviae* and 0.1006 *A.trixa*). The most optimal K for structure-like sNMF analysis was 3, and plots reveal that there is virtually no admixture present between the morphotypes, even in the populations at the sympatric sites (Fig. 3C).

Finally, to construct the phylogeny, we selected with ModelFinder a transversion substitution model of AG=CT and empirical unequal base frequencies, plus a FreeRate model (Yang 1995; Soubrier et al. 2012) for rate heterogeneity across sites with 2 categories, and an ascertainment bias correction appropriate for SNP alignments (TVM+F+ASC+R2). The output tree supports the pattern observed in the results above, that the morphotypes are consistently distinct, and that variation is lower in *A.bigeloviae* (Fig. 3B). The tree also suggests that Type III is more closely related to *A.bigeloviae* than *A.trixa* is, which may mean that their shared gall morphological traits are ancestral.

### Taxonomy

#### Aciurina luminaria

Taxon classificationAnimaliaDipteraTephritidae

Baine sp. nov.

295A5DDC-3B1F-5800-89C7-CD9C1E974D42

https://zoobank.org/BC9BB3A4-E1CC-4793-AC8D-B8A4E779FFBF

##### Type material examined.

**Figure 4.**
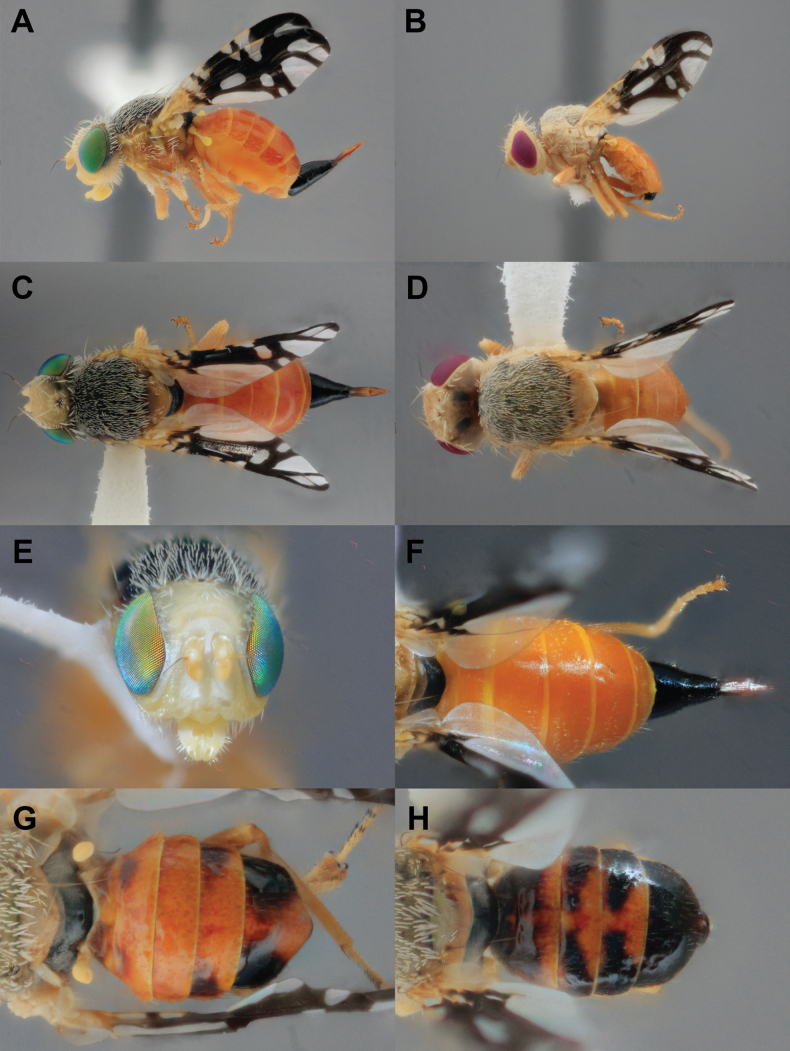
*Aciurinaluminaria* sp. nov. **A** holotype lateral habitus **B** allotype lateral habitus. Difference in eye color is a result of the age of mounted specimen **C** holotype dorsal habitus **D** allotype dorsal habitus **E** holotype head, anterior **F** holotype abdomen, dorsal **G–H** variation in dorsal abdomen color **G** mostly orange morph **H** dark morph.

***Holotype*** (Fig. 4A, C) USA • **NM**; *Santa Fe Co*.; ♀; Tesuque E arroyo crossing Road 74; 35.77686°N, 105.92904°W; 5 May 2021; Q. Baine leg.; MSBA81957. ***Allotype*** (Fig. 4B, D) USA • **NM**; *Santa Fe Co*.; ♂; 1 mi SE Chupadero off St Rd 592; 35.814°N, 105.907426°W; 7 May 2023; Q. Baine leg.; MSBA81946. ***Paratypes*** USA • **CO**; *Alamosa Co.*; 2♀ 1♂; S of Mosca side of Hwy 17; 37.62534°N, 105.86636°W; 20 May 2021; V. Martinson leg.; MSBA81880–81882 • 3♀ 3♂ 1 gall; San Luis State Wildlife Area, Lane 6 N; 37.66256°N, 105.72293°W; 20 May 2021; E. Martinson & V. Martinson leg.; MSBA81892–81893, MSBA81896–81897, USNMENT02011093–USNMENT02011095 • 4♀ 4♂; San Luis State Wildlife Area, Lane 6 N; 37.66256°N, 105.72293°W; 14 May 2023; Q. Baine leg. MSBA81926–81933 • 7♀ 6♂; W of San Luis Valley Regional Airport entrance, Alamosa; 37.4442°N, 105.86753°W; 14 May 2023; Q. Baine leg.; MSBA81898–81901, USNMENT02011096–USNMENT02011097 • 2♀ 3♂; 2 mi S Zapata Falls turnoff Hwy 150; 37.59092°N, 105.6015°W; 15 May 2023; Q. Baine leg.; MSBA81909–81913. 2♀ 1♂; Road N 110 and Lane 1 N off Hwy 17; 37.59092°N, 105.6015°W; 15 May 2023; Q. Baine leg.; MSBA81914–81916 • 2♀ 1♂; Corner of Cortez Rd and Van Iwarden Dr, Alamosa; 37.43771°N, 105.88511°W; 14 May 2023; Q. Baine leg.; MSBA81922–81925. *Chaffee Co.* • 2♀ 2♂; Hwy 285 W of Johnson Village; 38.80956°N, 106.11603°W; 20 May 2021; E. Martinson leg.; MSBA81883–81886 • **NM**; *Cibola Co.*; 2♀ 2♂; El Malpais National Conservation Area Narrows; 34.96499°N, 107.81464°W; 15 May 2022; V. Martinson leg.; MSBA81876–81879. *San Juan Co.* • 1♂; S Bloomfield Hwy 550 Kutz Wash; 36.64524°N, 108.00264°W; 18 March 2022; E. Martinson leg.; MSBA81891. *Sandoval Co.* • 4♀; N side Hwy 550, La Jara; 36.0581873°N, 106.9749619°W; 31 May 2020; D. Hughes leg.; MSBA81887–81890. *Santa Fe Co.* • 5♀ 4♂, 1 gall; Tesuque E arroyo crossing Road 74; 35.77686°N, 105.92904°W; 7 May 2023; Q. Baine leg.; MSBA81934–81938, MSBA81941–81942, USNMENT02011090–USNMENT02011092 • 2♀; Tesuque E arroyo crossing Road 74; 35.77686°N, 105.92904°W; 5 May 2021; Q. Baine leg.; MSBA81956–81957 • 4♀ 2♂; 1 mi SE Chupadero off St Rd 592; 35.814006°N, 105.907426°W; 7 May 2023; Q. Baine leg.; MSBA81943–81948 • 3♀ 4♂; Tesuque Arroyo Ancho and Meredith Dr on Tesuque Village Rd; 35.752991°N, 105.934346°W; 7 May 2023; Q. Baine leg.; MSBA81949–81955. *Taos Co.* • 2♀ 3♂; 3 mi N Ojo Caliente off Hwy 285; 36.33046°N, 106.00581°W; 14 May 2023; Q. Baine leg.; MSBA81917–81921 • **UT**; *Kane Co.*; 1♀ 1♂; Coral Pink Sand Dunes; 3 July 1966; E.J. Allen leg.; WFBM0050980–0050981.

##### Other material examined.

USA • **NM**; *Alamosa Co.*; 4 galls, 1 pupa; S Nageezi side of Pueblo Pintado Rd; 36.21480°N, 107.69633°W; 23 May 2024; S. Rollins leg • 4♀ 3♂; Corner of Cortez Rd and Van Iwarden Dr, Alamosa; 37.43771°N, 105.88511°W; 14 May 2023; Q. Baine leg. • 3 larvae; San Luis State Wildlife Area, Lane 6 N; 37.66256°N, 105.72293°W; 20 May 2021; E. Martinson leg.

##### Diagnosis.

This wing pattern of the adult *A.luminaria* can be distinguished most easily from both *A.bigeloviae* and *A.trixa* by the elongate hyaline spot in cell br, consistent dark brown region surrounding crossvein r-m, and lack of dark stripe in anal cell; it further from *A.bigeloviae* by lack of dark stripe in the postero-distal region of cell m and lack of medial dark stripe in cell cua_1_ (frequently present in *A.trixa* also). It differs from the similar-looking *A.maculata* (Cole, 1919) and *A.lutea* (Coquillett, 1899) by the hyaline cell bc and hyaline basal region of cell br. The extent of bright orange on the abdomen of many *A.luminaria* specimens also distinguishes it from *A.maculata* which has a more red abdomen, and from *A.bigeloviae* and *A.trixa* which frequently have a dark orange, brown, or black abdomen. Genitalia structures are highly similar to that of *A.bigeloviae*, except perhaps for the rounded tips of the prensisetae which differ from illustrations in Steyskal (1984). However, Steyskal describes *A.bigeloviae* (at the time synonymized with *A.trixa* and *A.semilucida*) as being highly variable in male terminalia characters, so this may or may not be reliably diagnostic. The gall can be distinguished from *A.bigeloviae* and *A.maculata* by the pointed, teardrop shape, and from all remaining galls in the genus by the thick layer of dense tomentum covering the surface (Fig. 2).

##### Description.

Female body length (minus terminalia) 6 mm.

***Head*** (Fig. 4E) uniformly pale yellow except for occiput and narrow interocular margin grey and moderately pilose. Compound eye bright green, drying to dull red. Three pairs of frontal setae, two pairs of orbital setae, and one pair of ocellar setae present. All setae pale yellow in color matching frons in color. Antenna yellow with black arista.

***Thorax.*** Scutum and dorsal portions of pleura dark gray in background color with pale gray pollinosity and dense pale yellow setulae making the scutum appear pale yellow-gray in color at a distance. Scutellum pale orange-brown at apex, narrowly gray at base. Subscutellum with anterior half pale yellow, posterior half and all of mediotergite black with pale gray pollinosity. Ventral part of pleura yellow-orange. The following setae are present, and pale yellow: basal scutellar, postalar, intra-alar, acrostichal, postsutural dorsocentral, presutral supra-alar, postsutural supra-alar, two notopleural, postpronotal, anepisternal, and katepisternal. Anepimeral seta indistinguishable from surrounding setulae. Legs wholly orange in color except for black tarsal claw and apical tarsal setae. Forefemur with elongate comb-like setae. Wing 4.2 mm in length. Costa pale orange. Setae narrowly present dorsally at junction of R_2+3_ and vein R_4+5_. Wing coloring is dark brown to black with the following hyaline regions: cell bc, base of cell br, two vertical bands in cell c, the proximal one extending posteriorly halfway into cell bm, two marginal spots in r_1_ with apical spot extending into r_2+3_, large (2× wide as high) subapical spot in cell br, medial spot in cell bm, large basal and small apical spot in cell cua_1_, subapical spot (1.5× high as wide) in cell dm, entire cell cup except for narrowly at apex, alula, anal lobe, large basal marginal spot in cell m, and subapical band extending from posterior margin in cell m into cell r_4+5_ reaching vein R_4+5_. Halteres bright yellow.

**Figure 5.**
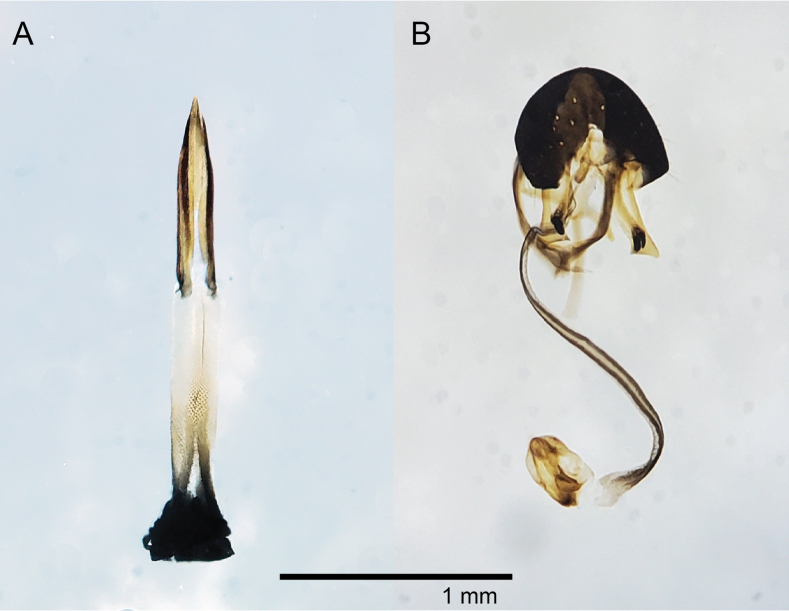
*Aciurinaluminaria* terminalia **A** female **B** male.

***Abdomen*** bright red-orange and shiny. Oviscape wholly black and shining. Eversible membrane brown, with shallowly semicircular cuticle denticles. Aculeus short (0.8 mm), notched at basal edge. Apical one third of aculeus with minute denticles covering medial edge (Fig. 5A).

Male body length (minus terminalia) 4 mm. Matching female in all respects except for terminalia. Epandrium black and shining, and proctiger pale yellow-orange. Surstylus pale brown, and prensisetae paired, bluntly rounded at the tips, and black. Phallus (1.25 mm long) and glans dark brown (Fig. 5B).

***Variation.*** Ventral thoracic pleura (including episternum, meron, anatergite and katatergite) in darker morphs are black with gray pollinosity, as on the scutum. Abdomen color ranges from wholly orange, orange with black tergite 6 (5 in male), orange with lateral black spots on tergites 5 and 6 (4 and 5 in male), to mostly black with orange background in dark morphs of both sexes (Fig. 4F–H).

***Immature.*** Second instar larva: Body white, elliptical-oblong and rounded on both anterior and posterior ends. Body segmented by rows of acanthae. Gnathocephalon conical and generally smooth. Mouth hook black and bidentate. Posterior spiracular plate with three pale brown rimae. Puparium: length 4.00 mm, width 1.62 mm. Dark brown, shining, elliptical-oblong, and rounded on both anterior and posterior ends. Anterior end with invagination scar and anterior thoracic spiracle. Posterior spiracular plate with spiracle darkened and flat.

Gall relatively large at maturity (7.24 mm mean latitudinal diameter), has a mostly rounded oblong to tapered teardrop shape and is covered uniformly in dense off-white cottony tomentum (Fig. 2C).

##### Biology.

*Aciurinaluminaria* is univoltine and has a life cycle and phenology similar to *A.bigeloviae* and *A.trixa* (Baine et al. 2023a; 2023b). Eggs are laid singly into the leaf bud of a distal plant stem. The gall forms at the oviposition site and the developing larva feeds on the tissue surrounding the central chamber of the gall. By fall, the gall reaches full size, and the larva reaches its final instar and chews to the outer layer of the gall to create a circular trap door. The larva ceases feeding and overwinters inside the gall, then pupates in the spring. Adults eclose in summer and push their way through the door to emerge from the gall and find a mate. The period of emergence from galls reared by these authors is May 29^th^ – June 28^th^. The latest date of emergence in examined material from Utah is July 18^th^ (gall collected 3 July 1966).

##### Associated arthropods.

The most common parasitoid by far is *Eurytomachrysothamni* (Hymenoptera: Eurytomidae). We reared very few unidentified *Halticoptera*, *Pteromalus* (Pteromalidae), and *Torymus* (Torymidae) wasps, which may be the same species as those associated with *A.bigeloviae* (Baine et al. 2023a). We also observed and reared a small number of *Rhopalomyia* (Diptera: Cecidomyiidae) hypergalls on the surface of galls collected in northwestern New Mexico. The hypergall system has been previously documented on both *A.bigeloviae* (Baine et al. 2023b) and *A.trixa* (Russo 2021), but whether the midge species is the same is unknown. Unexpectedly, and unknown from other *Aciurina* systems, a single *Synergus* (Hymenoptera: Cynipidae) wasp was also reared.

##### Host plant.

The known host plant is strictly Ericamerianauseosasubsp.ammophila L.C. Anderson, which was described from the San Luis Valley in Colorado (Anderson 2006). This plant is restricted to sandsheet and sand dune habitat and is known from southern Colorado (Anderson 2006) and here we add to its range northern New Mexico. Floral specimens from galled plants in New Mexico are deposited in MSB Herbarium (UNM0143677–UNM0143682). The host plant of material from Pink Sand Dunes, Utah is only identified as “*Chrysothamnusnauseosus*” [sic] and the host plants of gall observations in Arizona are unidentified.

##### Geographical range.

Beyond the localities of the examined material above, we have confirmed the presence of this species in some locations reported by Dodson and George (1986): Great Sand Dunes National Monument, CO, and near the cities Grants and Gallup, NM. We are aware of a specimen collected from Kanab, UT (A. Norrbom, pers. comm. Aug 2024). We are also able to definitively identify from photos the distinctive tomentum and shape of this gall on iNaturalist. Thus, the following localities are added to our own observations to the range of *A.luminaria* from public user observations: Petrified Forest National Park, AZ (obs. no. 2848593 & 112933554); Porcupine Spring, AZ (170773584); Brown’s Canyon, CO (151317053); Nageezi, NM (141568708); Aztec, NM (151553769); White Sands National Park, NM (199614395); and Kodachrome Basin State Park, UT (57095918).

##### Etymology.

The species epithet is a noun derived from the Spanish word for “light” which is specifically used in the southwest United States for small decorative lanterns traditionally displayed during the winter leading up to Christmas. We chose this epithet because the shape of this species’ gall is similar to that of a small flame on a candle, like those inside luminarias. Furthermore, this species’ galls are easiest to find when they are mature, and after the host leaves have dropped, so they are also associated with display in wintertime in the Southwest. The tradition of luminarias is common and adored in New Mexico, the type locality of this species. We elected to use the more widespread term luminaria over northern New Mexico regionally specific “farolito” because the species’ range extends into other regions in the West.

## Discussion

We used multiple lines of evidence to illuminate the species boundaries in an oft-confused complex of gall-inducing flies in the southwestern United States. *Aciurinaluminaria* induces galls of a distinct and diagnostic shape on a different *E.nauseosa* subspecies than its sympatric relatives, *A.bigeloviae* and *A.trixa*. It can further be consistently separated from these species by consistent differences in the adult wing pattern, and by genotyping via reduced representation genome sequencing.

We provide the following supplement, modified from that of Headrick et al. (1997) couplets to modify the key to species of *Aciurina* by Foote et al. (1993), with figure citations referencing Foote et al. (1993) except as noted. We have also removed a character from the key of Headrick et al. (1997: 419) that we found to be inconsistently present in both *A.bigeloviae* and *A.trixa* in New Mexico, and therefore not reliable as a diagnostic character: “pterostigma of at least one wing with a proximal hyaline or subhyaline incision”.

### Additions to the key to species of *Aciurina* by Foote et al. (1993)

**Table:** 

10	Pterostigma along costa no more than 1.5× as long as its greatest width (fig. 121c); vein dm-cu nearly straight (fig. 121e), the lower apical angle of cell dm ~ 65° (fig. 121f); wing predominantly hyaline	***A.notata* (Coquillett)**
–	Pterostigma along costa at least 2.0 × as long as its greatest width (fig. 124a); vein dm-cu usually bowed apicad (fig. 124b), the lower apical angle of cell dm seldom less than 90° (fig. 124c); wing with approximately equal area hyaline and brown, or predominantly brown	**11**
11	Proximal marginal hyaline incision in cell m lacking median, dark mark	**12**
–	Proximal marginal hyaline incision in cell m with a median, dark, often elongate mark (fig. 122), which sometimes divides the incision (Steyskal 1984: fig. 13) (Dodson and George 1986: fig. la, b); galls spheroid with cottony tomentum	***A.bigeloviae* (Cockerell)**
12	Anal cell bisected at least partially by medial brown mark from veins A1+CuA1 extending posteriorly, often reaching posterior wing margin; hyaline spot within cell br of the wing subcircular, 1–1.5 × as long as wide; brown region surrounding vein r-m paler in color than remaining dark part of wing, appearing like a diffuse orange spot; submedial dark mark usually present crossing cell cua_1_ from vein CuA_1_ to posterior wing margin; galls without tomentum	***A.trixa* Curran**
–	Anal cell without medial brown mark; hyaline spot within cell br of the wing elongated longitudinally, 1.5–2.5 × as long as wide; brown region surrounding vein r-m consistent in color, no diffuse spot present; submedial dark mark in cell cua_1_ absent; galls frequently ovoid or teardrop shaped with dense cottony tomentum	***A.luminaria* Baine, sp. nov.**

The adaptive significance of melanized wing patterns present on tephritid fly species is unclear as studies have found evidence that these patterns could play a role in sexual communication (Benelli et al. 2014; Hippee et al. 2022), thermoregulation (Sivinski and Pereira 2005), or predator deterrence by salticid mimicry (Mather and Roitberg 1987; Whitman et al. 1988; Rao and Díaz‐Fleischer 2012). From this study, it is clear that *A.luminaria* wings are not sexually dimorphic, similar to *A.bigeloviae* and *A.trixa*. However, it is also clear that *A.luminaria* wings have less melanized area on them than these species, and in combination with their usually paler abdomens, indicates that there may be an advantage to paler colors in their habitats.

The lower nucleotide diversity of *A.luminaria* suggests it is a more recently speciated group, and potentially the result of a “founder-effect” in which very few individuals from a population establish a lineage after colonizing a novel niche on a different host plant variety (Balakrishnan and Edwards 2009). This is supported by our observations of wing pattern variation, which is higher in *A.bigeloviae* and lower in *A.luminaria*. However, in the field, populations of the host plant E.n.subsp.ammophila appear largely fragmented across the range, often separated by dozens of miles, so the dispersal mechanisms of this species, if it was evolved from a single founding population, is mysterious.

Because *A.luminaria* occurs in sympatry with *A.bigeloviae* but on a unique host plant subspecies, speciation may be a result of host-race formation. Evidence of speciation via host-race formation, from a host switch specifically, is well-supported in the tephritid genus *Rhagoletis*, who display extraordinary host fidelity similar to that observed in *Aciurina* (Abrahamson and Blair 2008). Host-race formation is also documented in the closely related galling tephritid *Eurostasolidaginis* (Fitch, 1855), where two populations that are specific to distinct species of host *Solidago* (goldenrod) are reproductively isolated via assortative mating, and oviposition preference to the plant species of maternal provenance (Abrahamson and Blair 2008). Though we do not have documentation of *A.luminaria* mating or ovipositional behavior, the lack of intermediates in both our wing and genomic analyses suggest a similar level of isolation that may be maintained by similar barriers. In both *Eurosta* and *Rhagoletis*, there is substantial evidence that host switches were advantageous for enemy escape (Brown et al. 1995; Feder et al. 1995); a new niche, like a host plant and/or altered gall form may be absent of, or inaccessible to, predators that previously had a negative impact on fitness of the fly. In rearing *A.luminaria* for this description, we observed many fewer enemy parasitoids than have been reared from *A.bigeloviae* in a similar and overlapping range (Baine et al. 2023a), suggesting a lower rate of attack and therefore the possibility of enemy escape driving differentiation.

Although evolutionary biologists frequently view species delimitation as an impossible task due to disagreement on significant characters that define species concepts (De Queiroz 2007), we can describe species with relatively high confidence if we use “integration by congruence” which delimits based on multiple, independent, taxonomic characters (e.g., ecological niche + DNA) (Padial et al. 2010). In the case of *A.luminaria*, we employ integrative taxonomy to use the combined evidence of distinction in ecological niche, diagnostic morphology, and genomic structure to recognize a new species.

## Supplementary Material

XML Treatment for Aciurina luminaria

## Additional information

### Author ORCIDs

Quinlyn Baine https://orcid.org/0000-0001-5025-3741

Vincent G. Martinson https://orcid.org/0000-0001-5824-3548

Ellen O. Martinson https://orcid.org/0000-0001-9757-6679

### Data availability

All of the data that support the findings of this study are available in the main text or Supplementary Information. Sequence data used in phylogenetic analysis is stored in NCBI GenBank within BioProject ID PRJNA1075688.

## Supplementary materials

Supplementary material 1 Selection of mounted wing pairs from each of the three sampled morphotypes to show variation in wing pattern Data type pdf https://binary.pensoft.net/file/1149328This dataset is made available under the Open Database License (http://opendatacommons.org/licenses/odbl/1.0/). The Open Database License (ODbL) is a license agreement intended to allow users to freely share, modify, and use this Dataset while maintaining this same freedom for others, provided that the original source and author(s) are credited.Quinlyn Baine, Branden White, Vincent G. Martinson, Ellen O. Martinson
