# Supplementary material for: Discovery of a new gall-inducing species, Aciurinaluminaria (Insecta, Diptera, Tephritidae) via multi-trait integrative taxonomy
Source: Zookeys. 2024 Oct 7;1214:217–36. doi: 10.3897/zookeys.1214.130171 (PMC11491733; doi:10.3897/zookeys.1214.130171)
Supplement: Supplementary material 1 — Selection of mounted wing pairs from each of the three sampled morphotypes to show variation in wing pattern [file zookeys-1214-217_article-130171__-s001.pdf]

Supplement to: Discovery of a new gall-inducing species, *Aciurina luminaria* (Insecta, Diptera, Tephritidae) via multi-trait integrative taxonomy

Quinlyn Baine, Branden E. White, Vincent G. Martinson, Ellen O. Martinson. All: Department of Biology, 219 Yale Blvd, University of New Mexico, Albuquerque NM 87131

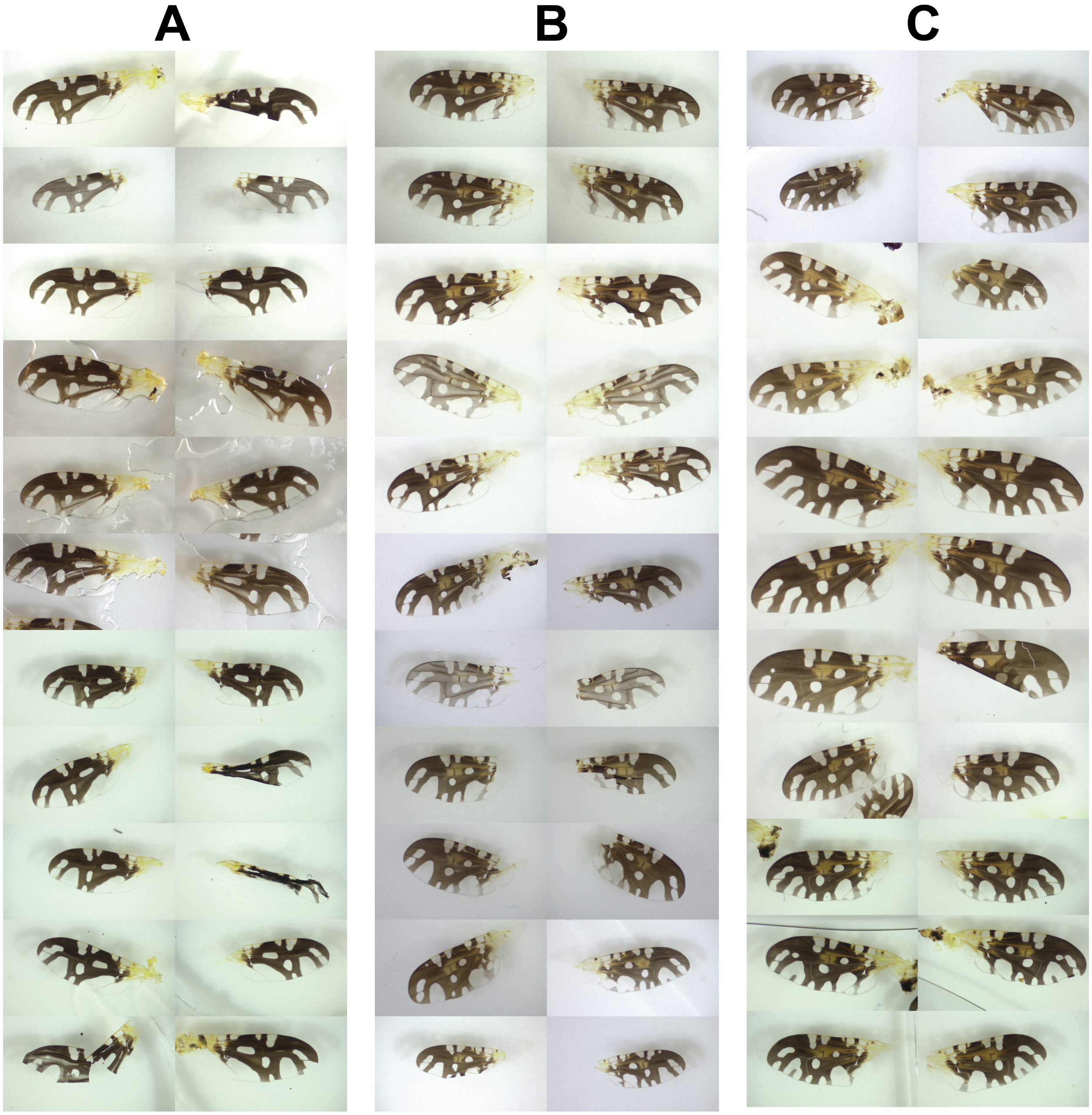

Supplementary Figure 1. Selection of mounted wing pairs from each of the three sampled morphotypes to show variation in wing pattern. A. *Aciurina luminaria*. B. *A. trixa*. C. *A. bigeloviae.*
